# A comparative study on the results of agonist and antagonist protocols based on serum AMH levels in patients undergoing intracytoplasmic sperm injection

**Published:** 2016-12

**Authors:** Fatemeh Nikmard, Behrouz Aflatoonian, Elham Hosseini, Abbas Aflatoonian, Mehrdad Bakhtiyari, Reza Aflatoonian

**Affiliations:** 1 *Anatomy Department, School of Medicine, Iran University of Medical Sciences, Tehran, Iran.*; 2 *Stem Cell Biology Research Center, Yazd Reproductive Sciences Institute, Shahid Sadoughi University of Medical Sciences, Yazd, Iran. *; 3 *Department of Advanced Medical Sciences and Technologies, School of Paramedicine, Shahid Sadoughi University of Medical Sciences, Yazd, Iran.*; 4 *Department of Obstetrics and Gynecology, Research and Clinical Center for Infertility, Shahid Sadoughi University of Medical Sciences, Yazd, Iran.*; 5 *Department of Endocrinology and Female Infertility at Reproductive Biomedicine Research Center, Royan Institute for Reproductive Biomedicine, ACECR, Tehran, Iran.*

**Keywords:** *AMH*, *Ovarian stimulation protocols*, *Oocyte*, *Embryo*, *Pregnancy rate*

## Abstract

**Background:**

Serum concentrations of antimullerian hormone (AMH) correlate with ovarian response during assisted reproduction treatment (ART) cycles.

**Objective:**

This retrospective study attempted to evaluate the selection of ovarian stimulation protocols based on serum AMH levels in patients and its impact on the results of ART.

**Materials and Methods:**

Based on AMH levels, the patients with tubal factor infertility were divided in three groups of normal, low and high AMH levels. Oocyte, good embryo number and pregnancy rate in each group were analyzed.

**Results:**

Using agonist and antagonist protocols, an increase in serum AMH led to higher number of oocytes and better quality embryos. At all low, normal and high AMH levels, the agonist protocol led to a more significant increase in the number of oocytes than the antagonist protocol (p<0.05). The number of high quality embryos significantly increased by the agonist protocol than antagonist protocol in women with normal AMH levels of 1.3-2.6 ng/ml (p=0.00). Moreover, the results for the number of high quality embryos at AMH ˃2.6 ng/ml was in favor of the antagonist protocol (p=0.00). The results showed the lowest pregnancy rate at AMH ˂1.3 ng/ml. At AMH ˃2.6 ng/ml, there was a significant increase in pregnancy rate through the antagonist protocol (p=0.04).

**Conclusion:**

Findings of this study suggested that the ART results are predictable, taking into account the AMH levels. The protocol specific to each patient can be used given the AMH level in each individual. This is because the results of each protocol depend on individual conditions.

## Introduction

(COH) plays an important role in reproductive medicine. That is why the selection of an appropriate ovarian stimulation strategy can improve the results of assisted reproductive techniques. Although the two common ovarian stimulation protocols (GnRH agonist and GnRH antagonist) exhibit similar implantation and pregnancy rates, each protocol entails specific characteristics. In fact, the agonist protocol leads to higher number of oocytes per cycle, whereas the antagonist protocol curtails the gonadotropin dose, shortening the stimulation period and saving the treatment costs, which in turn leads to patient comfort (1-4). 

Therefore, a suitable treatment strategy should be selected according to the ovarian response required for each person by taking the patient’s conditions into account. Anti-mullerian hormone (AMH) is a predictor of ovarian response used to select the agonist and antagonist protocols. This can maximize the success rate of assisted reproductive techniques, while enhancing the safety of ovarian stimulation practices (5). AMH is a subcategory of TGFβ produced by the granulosa cells of pre-antral and antral follicles (6). AMH can prevent the growth and recruitment of primordial follicles by reducing the sensitivity of follicles to FSH (7). The direct relationship between serum AMH levels and the number of antral follicles has been demonstrated (8). 

Furthermore, the AMH levels do not fluctuate during the menstrual cycle (9, 10). AMH is considered as an indicator that can be employed as the best option during assisted reproductive treatment (11). There are very few studies on the application of AMH to predict the quality of oocytes and embryos (12). Based on the AMH level in each patient, the COH can improve the clinical pregnancy rate and minimize the harm associated with ovarian response (13). According to previous studies, different results have been obtained by using agonist and antagonist protocols in normal, low and high AMH levels (14). The COH may give rise to certain problems due to different ovarian responses to FSH (15). 

This extensive study intended to compare the number of oocytes, quality of embryo and ultimately pregnancy rate through the agonist and antagonist protocols in patients with different AMH levels. There have been no studies so far comparing the results of both protocols in normal, low and high responders based on serum AMH levels.

## Materials and methods

This retrospective study was conducted on 243 patients under ICSI treatment in the private assisted reproduction (Laleh Hospital) ICSI during 2012-2014.

**Characteristics**

The patients included women with tubal factor infertility at age less than 40 years, normal menstrual cycle, presence of two ovaries, with no history of ovarian surgery, chemotherapy, endocrine disorders and hormonal therapy. The preliminary analysis involved infertility details, medical history, past surgeries and obstetric examinations one month prior to the treatment. Furthermore, the trans-vaginal ultrasonography was performed to assess the local pathology and hormonal profile, including FSH, TSH and serum AMH levels in 2-3 days of menstrual cycle. 

**Ovarian stimulation protocols**

According to clinical diagnosis, the patients underwent ovarian stimulation through GnRH agonist and antagonist protocols. The GnRH agonist protocol involved daily subcutaneous injection of 0.1 mg triptorelin acetat (decapeptyl, Ferring Pharmaceuticals, Netherlands) in the mid-luteal phase (day 20) and menstrual cycle before the stimulation. The ovarian stimulation was obtained from day 2 or 3 of the cycle by subcutaneous injection of recombinant human FSH (Gonal-F, Merk Serono, Germany) at a fixed dose of 150-225 IU and then continued by injection of hCG. 

The ovarian response was monitored through serial ultrasonography and evaluation of serum estradiol levels. GnRH antagonist protocol performed by administration of gonadotropin rFSH on day 2 of the cycle along with GnRH antagonist cetrorelix (Cetrotide, ASTA Medica, Amsterdam, The Netherlands; 0.25 mg/d, s.c) on day 9 post-stimulation. In all patients, the ovulation stimulation was done with 5000-10000 units of hCG, leading to a follicle diameter of 19 mm and a desirable level of serum estradiol. The oocytes were retrieved through transvaginal ultrasound-guided technique 36 hours after administration of hCG. 

The intracytoplasmic sperm injection was performed following the oocyte retrieval and oocytes denudation. The incidence of fertilization was assessed 19-21 hr after the injection in the presence of two pronuclei. The quality of embryo was recorded based on the number of blastomeres and percentage of fragmentation 42-44 hr after the injection, which was then comparatively studied. This study attempted to compare the number of high quality embryos characterized by (i) 4 or 5 blastomeres on the 2^nd^ and minimum 7 blastomeres on the 3^rd^ day after fertilization, (ii) absence of multi-nucleotide blastomeres, and (iii) fragmentation of less than 20% on 2^nd^ and 3^rd^ days after fertilization. 

During the assessment of results, the patients were divided on the basis of serum AMH into three groups: 1) AMH ˂1.3 ng/ml, 2) AMH between 1.3-2.6 ng/ml and 3) AMH ˃2.6 ng/ml (14). This study exclusively compared the number of oocytes, high quality embryos and pregnancy rates using two different protocols at three AMH levels.

**Statistical analysis**

SPSS software (SPSS, version 21 for windows; SPSS Inc., Chicago. IL), ANOVA including Tukey internal test was used to compare quantitative variables between the groups and ^2^for qualitative variables. p˂0.05 was considered statistically significant.

## Results

**Figure 1:**
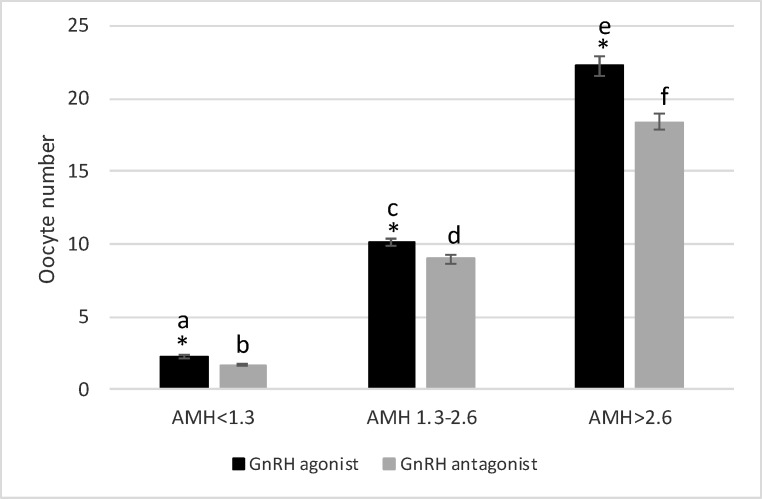
Oocyte retrieval in three AMH levels with GnRH agonist protocol vs antagonist protocol. *shows statistical difference between two protocols (p˂0.05). The letter shows statistical difference within and between groups (p˂0.05

**Figure 2:**
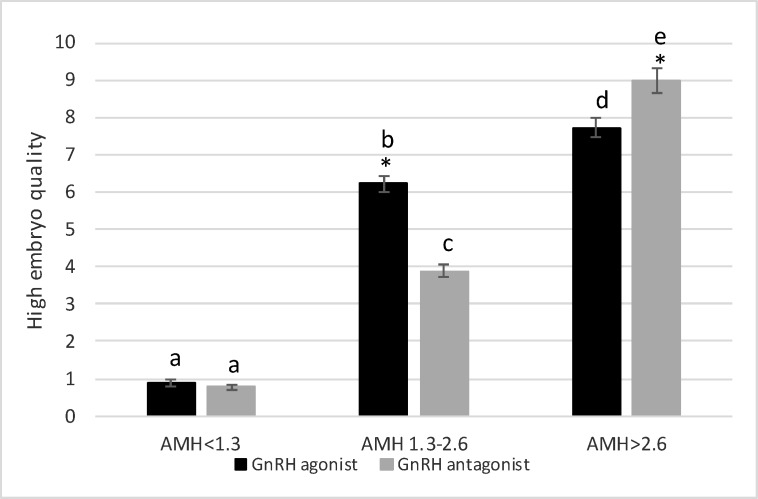
High embryo quality in three AMH levels with GnRH agonist protocol vs antagonist protocol. *shows statistical difference between two protocols (p˂0.05). The letter shows statistical difference within and between groups (p˂0.05

**Figure 3:**
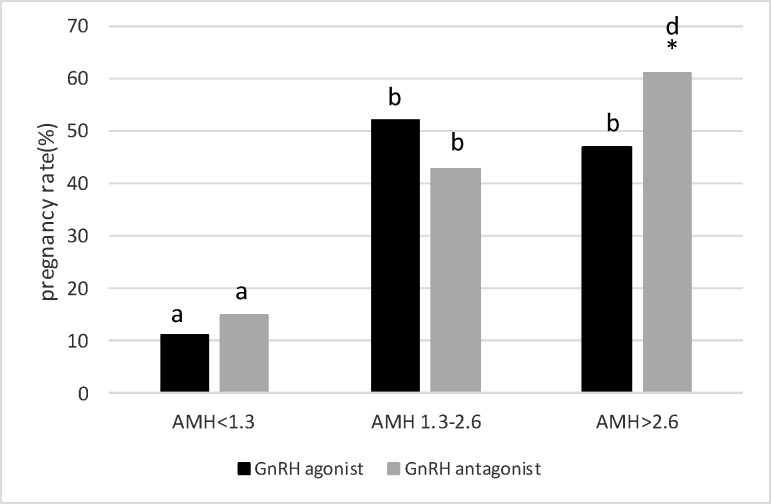
Pregnancy percentage in three AMH levels with GnRH agonist protocol vs antagonist protocol. *shows statistical difference between two protocols (p˂0.05). The letter shows statistical difference within and between groups (P˂ 0.05

The data on age and hormonal profiles have been listed in table I, where there are no significant differences between the two protocols in these three groups. The results indicated that the number of oocytes was significantly higher when using the agonist protocol in women with AMH of 1.3-2.6 ng/ml compared to the antagonist protocol (10.16±0.27 vs. 9.01±0.3, p=0.006) (Figure 1). The number of high quality embryos showed a dramatic increase by the agonist protocol than the antagonist protocol (6.23±0.2 vs. 3.88±0.17, p=0.00) (Figure 2). Despite the difference in the two protocols in the numbers of oocytes and high quality embryos, the pregnancy rates did not show any significant difference. Nonetheless, the pregnancy rate was higher in the agonist protocol than the antagonist protocol (52% and 43%, respectively) (Figure 3). 

In women with low AMH levels (˂1.3 ng/ml), the number of oocytes obtained by the agonist protocol was significantly higher than that by the antagonist protocol (2.23±0.11 vs. ˂2, p˂0.001) (Figure 1). Nevertheless, there was no significant difference between the agonist protocol (0.88±0.08) and the antagonist protocol (0.78±0.07) in the number of high quality embryos, p=0.34 (Figure 2). Although the number of oocytes and high quality embryos is in favor of the agonist protocol, the pregnancy rate in the antagonist protocol is higher than in the agonist protocol, indicating an insignificant difference (15% vs. 11%, respectively) (Figure 3).

In women with high ovarian response (AMH ˃2.6 ng/ml), there was a high number of oocytes obtained by agonist protocol, indicating a significant increase compared to antagonist protocol (22.26±0.64 vs. 18.41±0.55, p=0.00) (Figure 1). The comparison of the number of embryos showed different patterns. In fact, the number of high quality embryos was significantly higher when using the antagonist protocol, compared to the agonist protocol (9±0.32 vs. 7.71±0.26, p=0.002) (Figure 2). Similar to the number of high quality embryos, the pregnancy rate significantly increased when using the antagonist protocol, compared to the agonist protocol (61% vs. 47%, respectively) (Figure 3).

The comparison of AMH levels against the results of ovarian stimulation, quality of embryo and pregnancy rate demonstrated that an increase in AMH level significantly increased the number of oocytes and good quality embryos when using both protocols. In fact, the highest number of oocytes and high quality embryos in both protocols were observed at the highest AMH level, whereas, the lowest AMH level was associated with the lowest number of oocytes and embryos. The pregnancy rate patterns varied in different AMH levels in these two protocols. In fact, the highest pregnancy rate at normal AMH level was observed in agonist protocol, even though the difference was insignificant in comparison of high AMH level. 

**Figure 4:**
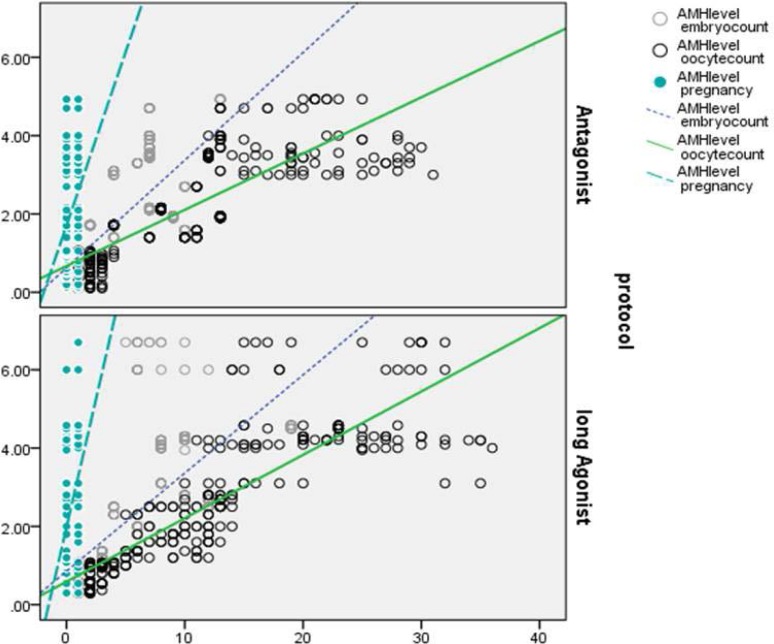
Correlations between AMH level, oocyte and embryo with positive pregnancy in both antagonist and agonist protocol.

**Figure 5:**
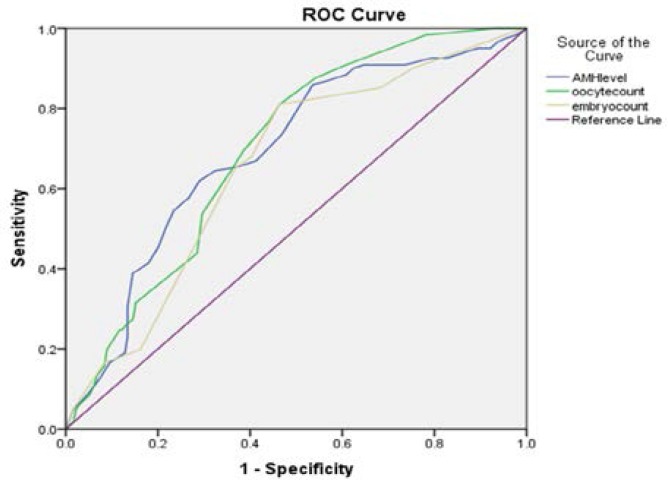
Comparison of predictive values for positive pregnancy using the ROC curve analysis in antagonist protocol

**Figure 6:**
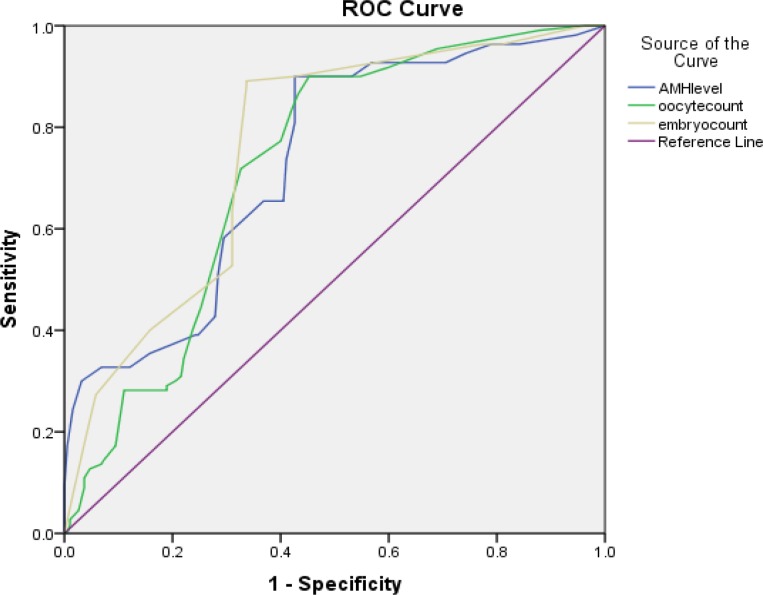
Comparison of predictive values for positive pregnancy using the ROC curve analysis in agonist protocol

The comparison of the normal and high AMH level groups, however, showed a significant increase in comparison of the lowest AMH level. In the antagonist protocol, the highest pregnancy rate was observed at the highest AMH level, indicating a significantly higher increase than the other two groups of AMH (1.3-2.6 and ˂1.3). Moreover, the pregnancy rate at normal AMH level increased significantly higher than the lowest level (˂1.3 ng/ml). In total, the levels of serum AMH in all patients undergoing antagonist protocol stimulation showed a positive correlation with the number of retrieved oocytes (r=0.82, p<0.01), embryo count (r=0.66, p<0.01) and pregnancy (r=0.32, p<0.01) (Figure 4). Also, the levels of AMH in patients who received agonist protocol showed a positive correlation with the number of retrieved oocytes (r=0.86, p<0.01), embryo count (r=0.68, p<0.01) and pregnancy (r=0.36, p<0.01) (Figure 1). Furthermore, according to the stimulation protocols used, AMH also was an accurate marker of pregnancy in both agonist and antagonist protocol (AUC of 0.69 and 0.72, respectively) (Figure 5, 6).

**Table I:** Mean (standard deviation) age, number of oocytes retrieved, embryo number, FSH, TSH levels in the GnRH-a long protocol vs. GnRH-ant protocol group

**Variables**		**GnRH-a long protocol** **(n = 123)**	**GnRH-ant protocol** **(n = 120)**	**p-value**
Age		36 ± 4.07	37 ± 3.6	0.11
Serum FSH (IU/L)				
	AMH <1.3 (ng/ml)	9.1 ± 0.45 (41)	8.58 ± 0.64 (39)	0.26
	AMH 1.3-2.6	6.65 ± 0.9 (42)	6.9 ± 0.61 (40)	0.33
	AMH>2.6	7.27 ± 0.71 (40)	7.61 ± 0.82 (41)	0.39
Serum TSH (IU/L)				
	AMH<1.3 (ng/ml)	2.66 ± 1.01	2.91 ± 0.98	0.79
	AMH 1.3-2.6	2.2 ± 0.34	2.67 ± 0.56	0.10
	AMH>2.6	2.83 ± 0.58	2.39 ± 0.43	0.68
BMI (kg/m^2^)				
	AMH<1.3 (ng/ml)	24 ± 1.5	23 ± 1.2	0.14
	AMH 1.3-2.6	22 ± 1.1	22 ± 1.17	0.81
	AMH>2.6	21 ± 1.2	22 ± 1.4	0.43

FSH: follicle-stimulating hormone

TSH: thyroid-stimulating hormone

AMH: anti-mullerian hormone

BMI: body mass index

## Discussion

In this study, the results of GnRH agonist and GnRH antagonist protocols were explored based on serum AMH levels. It was proven that an increase in serum AMH level led to a higher number of oocytes and embryos. These results were consistent with the findings of other studies. The serum AMH level can be adopted as a predictor of ovarian response in ART cycles (5, 16). Ovarian response is an important success factor in ART results. The two common protocols of ovarian stimulation are associated with different ovarian responses, each entailing a unique characteristic (17). 

Just as demonstrated in this study, the use of two different protocols in women with low serum AMH levels led to different ovarian responses. In this regard, some studies preferred the GnRH agonist, whereas others the GnRH protocol for patients with low AMH levels or a history of weak ovarian response (18, 19). 

According to the current study, although the GnRH agonist yielded a greater number of oocytes, it did not increase the number of high quality embryos compared to the GnRH antagonist. Moreover, the pregnancy rate was higher when the antagonist protocol was used. What ultimately matters in selecting the ovarian stimulation protocol is the outcomes. At first glance, the agonist protocol might indicate poorer results in this category of patients, but the outcomes of the antagonist protocol tended to be more satisfactory at the end. Similar studies which preferred the antagonist protocol in patients with low AMH levels indicate treatment costs decline and higher patients’ comfort, owing to a reduction in gonadotropin dose and canceled cycles. By the same token, a broader study has been conducted on ovarian stimulation protocol in women with low AMH levels. Despite more intense stimulation through the agonist protocol, there was no difference in the number of oocytes, canceled cycles and pregnancy rate through the antagonist protocol (20). 

Similar to our findings, the previous study introduced the antagonist protocol as the front line of treatment for people with low AMH levels (21). Contrary to our findings, Malmusi *et al*, Sun and Zhu preferred the agonist protocol due to a higher number of oocytes and embryos in women with low AMH levels (22, 23). Regardless of the type of protocol, there was a very low pregnancy rate in this category of patients. This could be due to defects in granulosa cells, which produce the AMH as mentioned earlier. The defects in the supporting cells decreased the quality of oocytes, which in turn led to lower implantation and pregnancy in women with low AMH levels (24, 25). 

According to our findings, the agonist protocol in women with normal AMH levels leads to a greater number of oocytes and embryos (2). Moreover, the pregnancy rate was higher than that in the antagonist protocol (26, 27). Some studies have suggested that the agonist protocol in patients with normal ovarian response is associated with higher number of oocytes, implantation and pregnancy rates, as well as an increase in live births and reduction in canceled cycles (28). A few studies prefer the agonist protocol over the antagonist protocol in normal respondents because of the negative impact of the latter on the performance of major endometrial proteins (29). In this regard, there have been other studies rejecting that statement on the grounds that endometrial receptivity in the antagonist protocol is similar to the natural endometrial cycle (30). According to our findings, however, the agonist protocol yielded more favorable results in women with normal AMH levels and ovarian responses. 

The previous studies have suggested mixed results on high levels of serum AMH and selecting the type of treatment protocol (31). In the selection of agonist protocol for ovarian stimulation in patients with high AMH levels, Nelson *et al* and Manno *et al* reported an increase in pregnancy rate. Moreover, it was accompanied by an increase in the incidence of ovarian hyperstimulation syndrome (OHSS) and canceled cycles (17, 32). In contrast to these results, the molecular studies on the oocytes, obtained through the antagonist protocol in patients with high AMH levels, indicated that the gene expressions of ATPase and *BMP15*, as two indicators of oocyte quality, enhanced through the antagonist protocol (4). Therefore, the antagonist protocol can be the more desirable option in high AMH cases. In an analysis, La Marca and Sunkara argued that the antagonist protocol in patients with high ovarian responses curtails the risk of OHSS, because the use of antagonist protocol depends on the patient’s endocrine conditions (14). Hence, it may lead to lower number of follicles during ovarian stimulation. Moreover, it entails a shorter treatment period and higher pregnancy rate than the agonist protocol (33). 

These results are consistent with our findings, where the number of high quality embryos and pregnancy rates significantly increased despite the lower number of oocytes obtained by the antagonist protocol. Therefore, the antagonist protocol is the more suitable option for patients with high serum AMH levels. According to a positive correlation of AMH levels with the number of retrieved oocytes, embryo count and pregnancy rate in both different protocols, AMH is an adequate predictor of ovarian response and ART outcomes. Further AMH-tailored protocol selection improved ART outcomes. These findings are similar to Hamdine *et al* findings (15). 

This retrospective study generally demonstrated that the agonist protocol is the more suitable option for the normal range of serum AMH given the number of oocytes and high quality embryos leading to higher pregnancy rate. Since low AMH levels provide no prognosis for ovarian response and pregnancy rate, the selection of a good protocol can improve the pregnancy rate in patients who barely respond to treatment due to excessive ovarian stimulation. 

Hence, the administration of high-dose gonadotropin and agonist protocol are not associated with improvement of outcomes. It is better to select the antagonist as the more ideal ovarian stimulation strategy for low AMH level given the higher pregnancy rate. Since the antagonist protocol in patients with high AMH levels leads to greater patient comfort and lower risks associated with ovarian hyper-stimulation and above all, yielding more desirable outcomes, it can be regarded as a safe technique for ovarian stimulation. 

## Conclusion

According to these results, it can be concluded that the ovarian stimulation protocol should be selected based on the patient’s’ specific characteristics. Moreover, the prediction of ovarian response and pregnancy rate can curtail the treatment costs and pave the way for successful assisted reproductive techniques.
